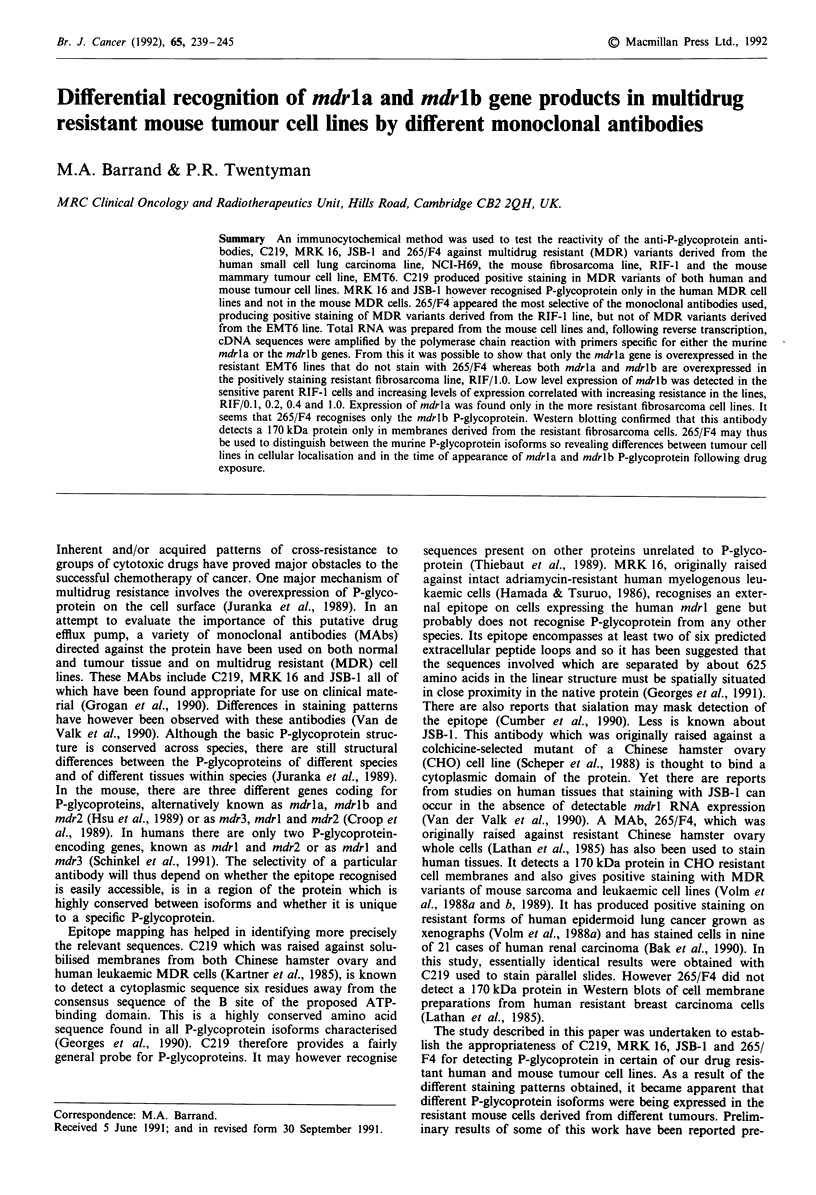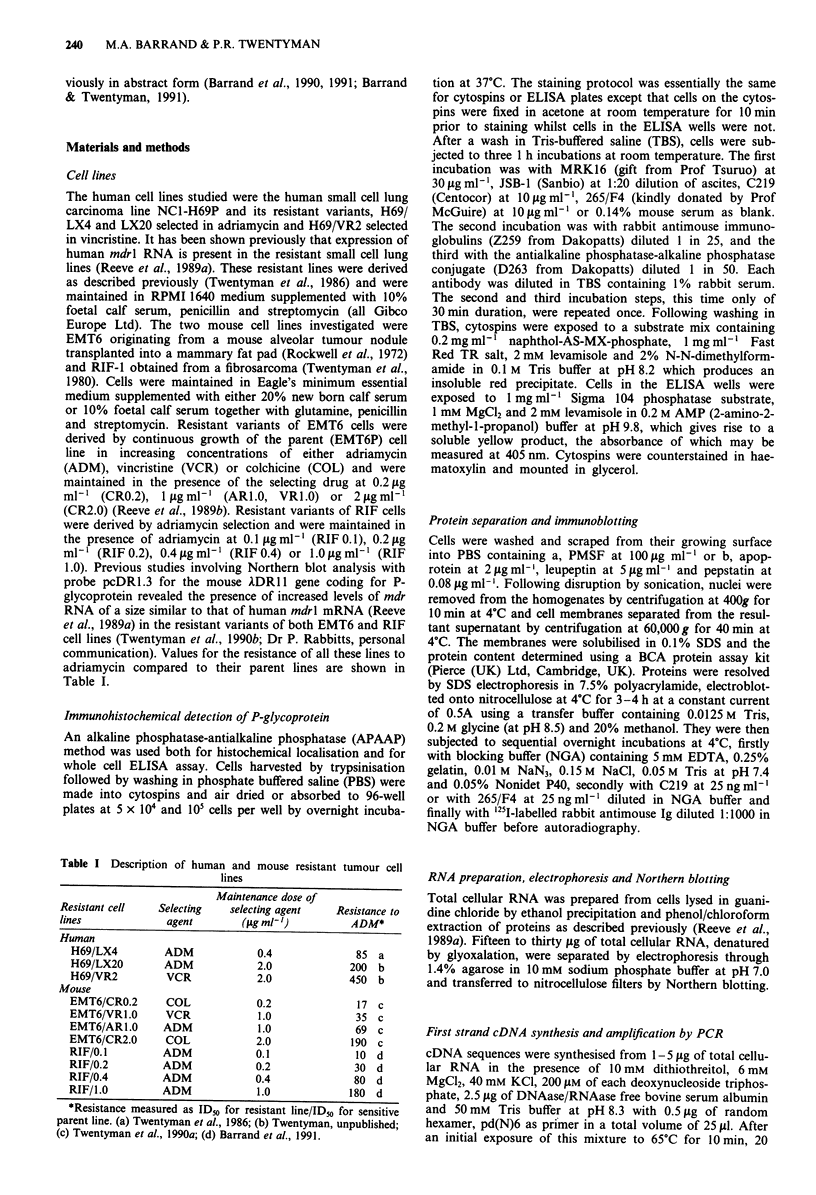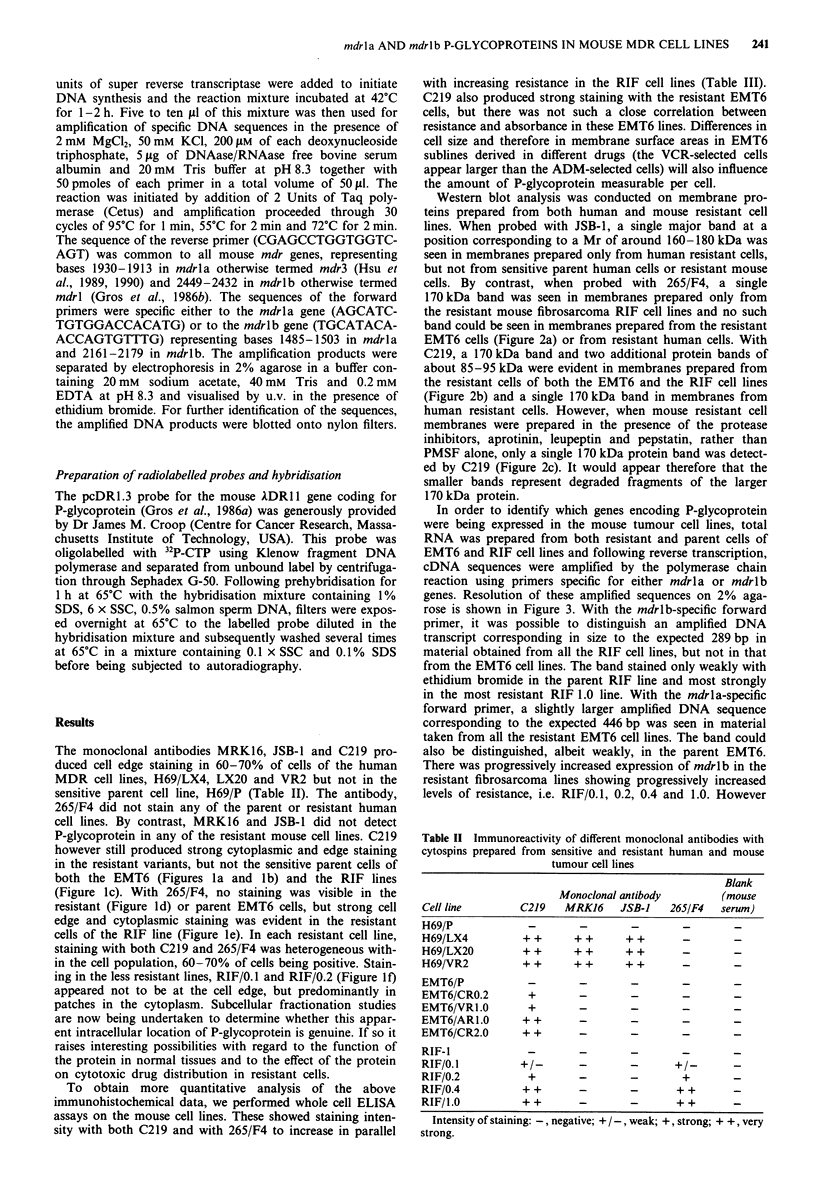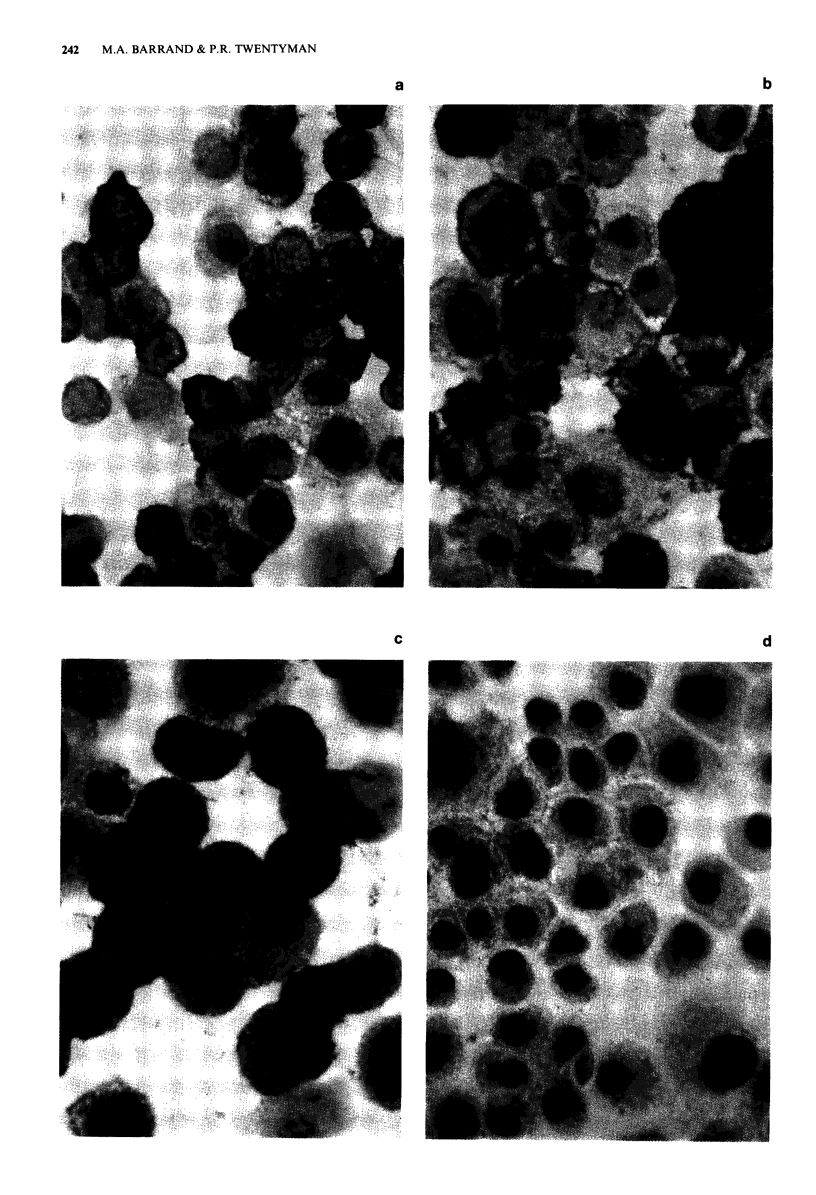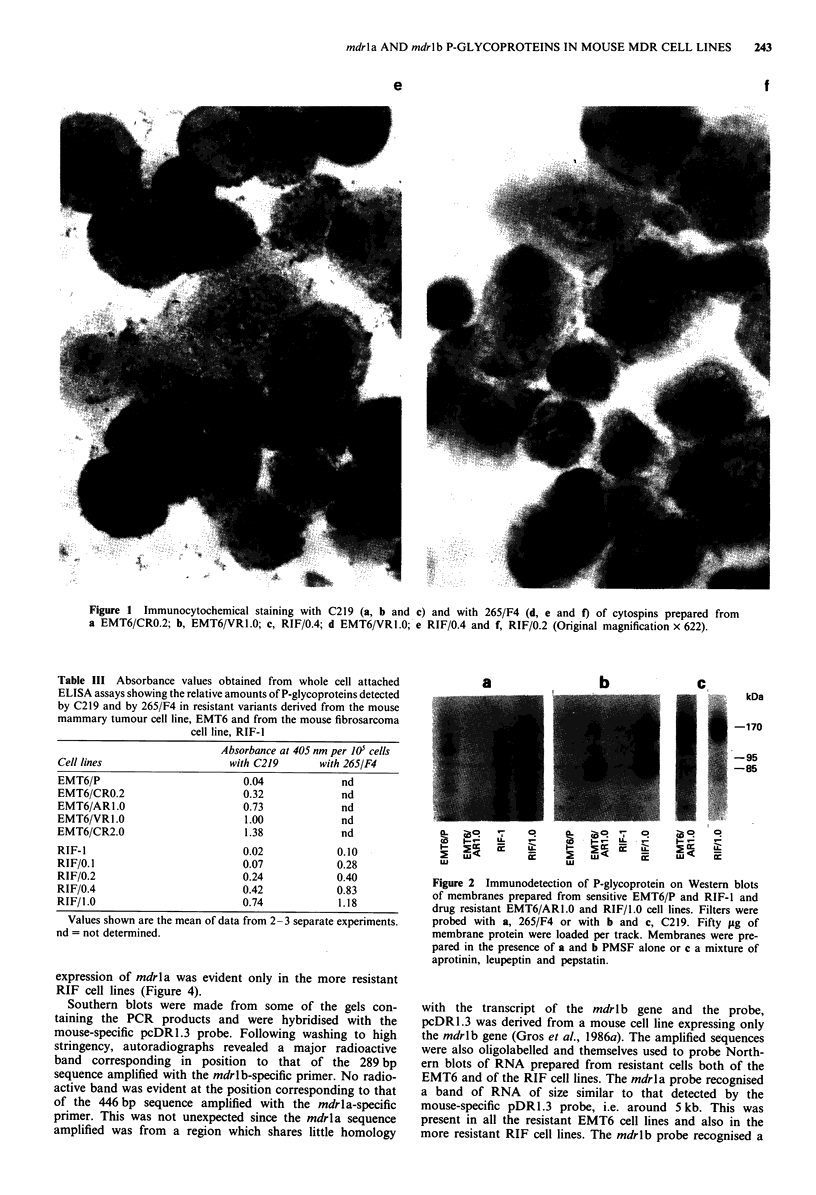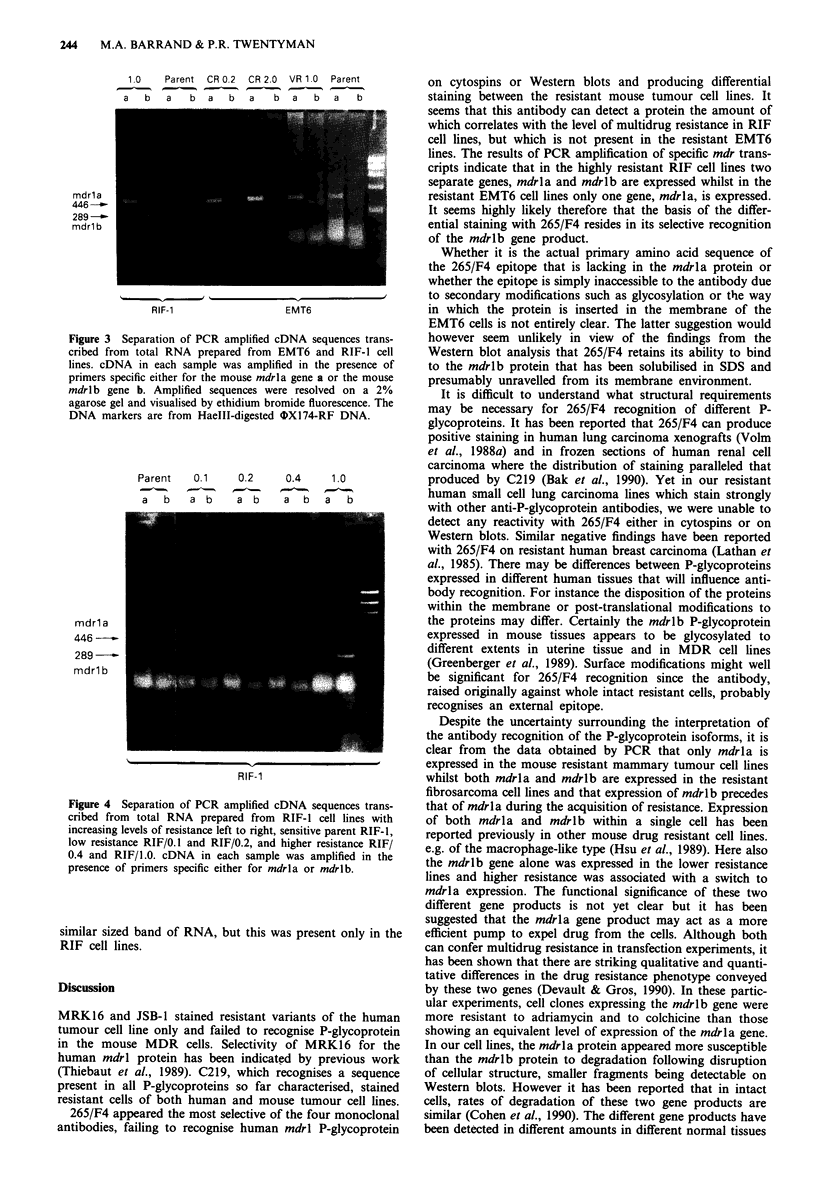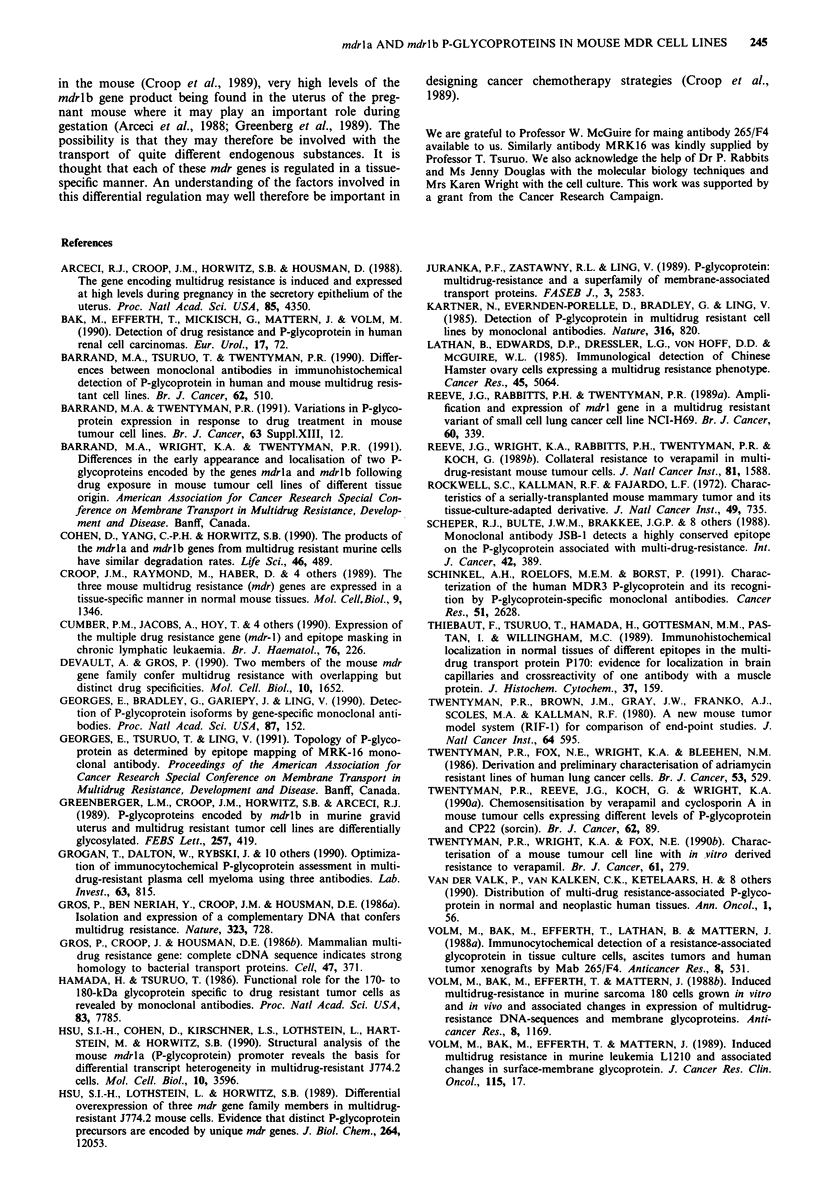# Differential recognition of mdr1a and mdr1b gene products in multidrug resistant mouse tumour cell lines by different monoclonal antibodies.

**DOI:** 10.1038/bjc.1992.48

**Published:** 1992-02

**Authors:** M. A. Barrand, P. R. Twentyman

**Affiliations:** MRC Clinical Oncology and Radiotherapeutics Unit, Cambridge, UK.

## Abstract

**Images:**


Br. J. Cancer (1992), 65, 239-245 © Macmillan Press Ltd., 1992

### Differential recognition of *mdr*1a and *mdr*1b gene products in multidrug resistant mouse tumour cell lines by different monoclonal antibodies

M.A. Barrand & P.R. Twentyman

*MRC Clinical Oncology and Radiotherapeutics Unit, Hills Road, Cambridge CB2 2QH, UK.*

**Summary** An immunocytochemical method was used to test the reactivity of the anti-P-glycoprotein antibodies, C219, MRK 16, JSB-1 and 265/F4 against multidrug resistant (MDR) variants derived from the human small cell lung carcinoma line, NCI-H69, the mouse fibrosarcoma line, RIF-1 and the mouse mammary tumour cell line, EMT6. C219 produced positive staining in MDR variants of both human and mouse tumour cell lines. MRK 16 and JSB-1 however recognised P-glycoprotein only in the human MDR cell lines and not in the mouse MDR cells. 265/F4 appeared the most selective of the monoclonal antibodies used, producing positive staining of MDR variants derived from the RIF-1 line, but not of MDR variants derived from the EMT6 line. Total RNA was prepared from the mouse cell lines and, following reverse transcription, cDNA sequences were amplified by the polymerase chain reaction with primers specific for either the murine *mdr*1a or the *mdr*1b genes. From this it was possible to show that only the *mdr*1a gene is overexpressed in the resistant EMT6 lines that do not stain with 265/F4 whereas both *mdr*1a and *mdr*1b are overexpressed in the positively staining resistant fibrosarcoma line, RIF/1.0. Low level expression of *mdr*1b was detected in the sensitive parent RIF-1 cells and increasing levels of expression correlated with increasing resistance in the lines, RIF/0.1, 0.2, 0.4 and 1.0. Expression of *mdr*1a was found only in the more resistant fibrosarcoma cell lines. It seems that 265/F4 recognises only the *mdr*1b P-glycoprotein. Western blotting confirmed that this antibody detects a 170 kDa protein only in membranes derived from the resistant fibrosarcoma cells. 265/F4 may thus be used to distinguish between the murine P-glycoprotein isoforms so revealing differences between tumour cell lines in cellular localisation and in the time of appearance of *mdr*1a and *mdr*1b P-glycoprotein following drug exposure.

Inherent and/or acquired patterns of cross-resistance to groups of cytotoxic drugs have proved major obstacles to the successful chemotherapy of cancer. One major mechanism of multidrug resistance involves the overexpression of P-glycoprotein on the cell surface (Juranka *et al.*, 1989). In an attempt to evaluate the importance of this putative drug efflux pump, a variety of monoclonal antibodies (MAbs) directed against the protein have been used on both normal and tumour tissue and on multidrug resistant (MDR) cell lines. These MAbs include C219, MRK 16 and JSB-1 all of which have been found appropriate for use on clinical material (Grogan *et al.*, 1990). Differences in staining patterns have however been observed with these antibodies (Van de Valk *et al.*, 1990). Although the basic P-glycoprotein structure is conserved across species, there are still structural differences between the P-glycoproteins of different species and of different tissues within species (Juranka *et al.*, 1989). In the mouse, there are three different genes coding for P-glycoproteins, alternatively known as *mdr*1a, *mdr*1b and *mdr*2 (Hsu *et al.*, 1989) or as *mdr*3, *mdr*1 and *mdr*2 (Croop *et al.*, 1989). In humans there are only two P-glycoprotein-encoding genes, known as *mdr*1 and *mdr*2 or as *mdr*1 and *mdr*3 (Schinkel *et al.*, 1991). The selectivity of a particular antibody will thus depend on whether the epitope recognised is easily accessible, is in a region of the protein which is highly conserved between isoforms and whether it is unique to a specific P-glycoprotein.

Epitope mapping has helped in identifying more precisely the relevant sequences. C219 which was raised against solubilised membranes from both Chinese hamster ovary and human leukaemic MDR cells (Kartner *et al.*, 1985), is known to detect a cytoplasmic sequence six residues away from the consensus sequence of the B site of the proposed ATP-binding domain. This is a highly conserved amino acid sequence found in all P-glycoprotein isoforms characterised (Georges *et al.*, 1990). C219 therefore provides a fairly general probe for P-glycoproteins. It may however recognise sequences present on other proteins unrelated to P-glycoprotein (Thiebaut *et al.*, 1989). MRK 16, originally raised against intact adriamycin-resistant human myelogenous leukaemic cells (Hamada & Tsuruo, 1986), recognises an external epitope on cells expressing the human *mdr*1 gene but probably does not recognise P-glycoprotein from any other species. Its epitope encompasses at least two of six predicted extracellular peptide loops and so it has been suggested that the sequences involved which are separated by about 625 amino acids in the linear structure must be spatially situated in close proximity in the native protein (Georges *et al.*, 1991). There are also reports that sialation may mask detection of the epitope (Cumber *et al.*, 1990). Less is known about JSB-1. This antibody which was originally raised against a colchicine-selected mutant of a Chinese hamster ovary (CHO) cell line (Scheper *et al.*, 1988) is thought to bind a cytoplasmic domain of the protein. Yet there are reports from studies on human tissues that staining with JSB-1 can occur in the absence of detectable *mdr*1 RNA expression (Van der Valk *et al.*, 1990). A MAb, 265/F4, which was originally raised against resistant Chinese hamster ovary whole cells (Lathan *et al.*, 1985) has also been used to stain human tissues. It detects a 170 kDa protein in CHO resistant cell membranes and also gives positive staining with MDR variants of mouse sarcoma and leukaemic cell lines (Volm *et al.*, 1988*a* and *b*, 1989). It has produced positive staining on resistant forms of human epidermoid lung cancer grown as xenographs (Volm *et al.*, 1988*a*) and has stained cells in nine of 21 cases of human renal carcinoma (Bak *et al.*, 1990). In this study, essentially identical results were obtained with C219 used to stain parallel slides. However 265/F4 did not detect a 170 kDa protein in Western blots of cell membrane preparations from human resistant breast carcinoma cells (Lathan *et al.*, 1985).

The study described in this paper was undertaken to establish the appropriateness of C219, MRK 16, JSB-1 and 265/F4 for detecting P-glycoprotein in certain of our drug resistant human and mouse tumour cell lines. As a result of the different staining patterns obtained, it became apparent that different P-glycoprotein isoforms were being expressed in the resistant mouse cells derived from different tumours. Preliminary results of some of this work have been reported pre-

Correspondence: M.A. Barrand.
Received 5 June 1991; and in revised form 30 September 1991.

viously in abstract form (Barrand *et al.*, 1990, 1991; Barrand & Twentyman, 1991).

### Materials and methods

### *Cell lines*

The human cell lines studied were the human small cell lung carcinoma line NCI-H69P and its resistant variants, H69/LX4 and LX20 selected in adriamycin and H69/VR2 selected in vincristine. It has been shown previously that expression of human *mdr*1 RNA is present in the resistant small cell lung lines (Reeve *et al.*, 1989*a*). These resistant lines were derived as described previously (Twentyman *et al.*, 1986) and were maintained in RPMI 1640 medium supplemented with 10% foetal calf serum, penicillin and streptomycin (all Gibco Europe Ltd). The two mouse cell lines investigated were EMT6 originating from a mouse alveolar tumour nodule transplanted into a mammary fat pad (Rockwell *et al.*, 1972) and RIF-1 obtained from a fibrosarcoma (Twentyman *et al.*, 1980). Cells were maintained in Eagle's minimum essential medium supplemented with either 20% new born calf serum or 10% foetal calf serum together with glutamine, penicillin and streptomycin. Resistant variants of EMT6 cells were derived by continuous growth of the parent (EMT6P) cell line in increasing concentrations of either adriamycin (ADM), vincristine (VCR) or colchicine (COL) and were maintained in the presence of the selecting drug at 0.2 $\mu$g ml$^{-1}$ (CR0.2), 1 $\mu$g ml$^{-1}$ (AR1.0, VR1.0) or 2 $\mu$g ml$^{-1}$ (CR2.0) (Reeve *et al.*, 1989*b*). Resistant variants of RIF cells were derived by adriamycin selection and were maintained in the presence of adriamycin at 0.1 $\mu$g ml$^{-1}$ (RIF 0.1), 0.2 $\mu$g ml$^{-1}$ (RIF 0.2), 0.4 $\mu$g ml$^{-1}$ (RIF 0.4) or 1.0 $\mu$g ml$^{-1}$ (RIF 1.0). Previous studies involving Northern blot analysis with probe pcDR1.3 for the mouse $\lambda$DR11 gene coding for P-glycoprotein revealed the presence of increased levels of *mdr* RNA of a size similar to that of human *mdr*1 mRNA (Reeve *et al.*, 1989*a*) in the resistant variants of both EMT6 and RIF cell lines (Twentyman *et al.*, 1990*b*; Dr P. Rabbitts, personal communication). Values for the resistance of all these lines to adriamycin compared to their parent lines are shown in Table I.

### *Immunohistochemical detection of P-glycoprotein*

An alkaline phosphatase-antialkaline phosphatase (APAAP) method was used both for histochemical localisation and for whole cell ELISA assay. Cells harvested by trypsinisation followed by washing in phosphate buffered saline (PBS) were made into cytospins and air dried or absorbed to 96-well plates at 5 × 10$^4$ and 10$^5$ cells per well by overnight incubation at 37°C. The staining protocol was essentially the same for cytospins or ELISA plates except that cells on the cytospins were fixed in acetone at room temperature for 10 min prior to staining whilst cells in the ELISA wells were not. After a wash in Tris-buffered saline (TBS), cells were subjected to three 1 h incubations at room temperature. The first incubation was with MRK16 (gift from Prof Tsuruo) at 30 $\mu$g ml$^{-1}$, JSB-1 (Sanbio) at 1:20 dilution of ascites, C219 (Centocor) at 10 $\mu$g ml$^{-1}$, 265/F4 (kindly donated by Prof McGuire) at 10 $\mu$g ml$^{-1}$ or 0.14% mouse serum as blank. The second incubation was with rabbit antimouse immunoglobulins (Z259 from Dakopatts) diluted 1 in 25, and the third with the antialkaline phosphatase-alkaline phosphatase conjugate (D263 from Dakopatts) diluted 1 in 50. Each antibody was diluted in TBS containing 1% rabbit serum. The second and third incubation steps, this time only of 30 min duration, were repeated once. Following washing in TBS, cytospins were exposed to a substrate mix containing 0.2 mg ml$^{-1}$ naphthol-AS-MX-phosphate, 1 mg ml$^{-1}$ Fast Red TR salt, 2 mM levamisole and 2% N-N-dimethylformamide in 0.1 M Tris buffer at pH 8.2 which produces an insoluble red precipitate. Cells in the ELISA wells were exposed to 1 mg ml$^{-1}$ Sigma 104 phosphatase substrate, 1 mM $MgCl_2$ and 2 mM levamisole in 0.2 M AMP (2-amino-2-methyl-1-propanol) buffer at pH 9.8, which gives rise to a soluble yellow product, the absorbance of which may be measured at 405 nm. Cytospins were counterstained in haematoxylin and mounted in glycerol.

**Table I** Description of human and mouse resistant tumour cell lines

| *Resistant cell lines* | *Selecting agent* | *Maintenance dose of selecting agent ($\mu$g ml$^{-1}$)* | *Resistance to ADM** | |
|---|---|---|---|---|
| *Human* | | | | |
| H69/LX4 | ADM | 0.4 | 85 | a |
| H69/LX20 | ADM | 2.0 | 200 | b |
| H69/VR2 | VCR | 2.0 | 450 | b |
| *Mouse* | | | | |
| EMT6/CR0.2 | COL | 0.2 | 17 | c |
| EMT6/VR1.0 | VCR | 1.0 | 35 | c |
| EMT6/AR1.0 | ADM | 1.0 | 69 | c |
| EMT6/CR2.0 | COL | 2.0 | 190 | c |
| RIF/0.1 | ADM | 0.1 | 10 | d |
| RIF/0.2 | ADM | 0.2 | 30 | d |
| RIF/0.4 | ADM | 0.4 | 80 | d |
| RIF/1.0 | ADM | 1.0 | 180 | d |

*Resistance measured as $ID_{50}$ for resistant line/$ID_{50}$ for sensitive parent line. (a) Twentyman *et al.*, 1986; (b) Twentyman, unpublished; (c) Twentyman *et al.*, 1990*a*; (d) Barrand *et al.*, 1991.

### *Protein separation and immunoblotting*

Cells were washed and scraped from their growing surface into PBS containing a, PMSF at 100 $\mu$g ml$^{-1}$ or b, apoprotein at 2 $\mu$g ml$^{-1}$, leupeptin at 5 $\mu$g ml$^{-1}$ and pepstatin at 0.08 $\mu$g ml$^{-1}$. Following disruption by sonication, nuclei were removed from the homogenates by centrifugation at 400*g* for 10 min at 4°C and cell membranes separated from the resultant supernatant by centrifugation at 60,000 *g* for 40 min at 4°C. The membranes were solubilised in 0.1% SDS and the protein content determined using a BCA protein assay kit (Pierce (UK) Ltd, Cambridge, UK). Proteins were resolved by SDS electrophoresis in 7.5% polyacrylamide, electroblotted onto nitrocellulose at 4°C for 3–4 h at a constant current of 0.5A using a transfer buffer containing 0.0125 M Tris, 0.2 M glycine (at pH 8.5) and 20% methanol. They were then subjected to sequential overnight incubations at 4°C, firstly with blocking buffer (NGA) containing 5 mM EDTA, 0.25% gelatin, 0.01 M $NaN_3$, 0.15 M NaCl, 0.05 M Tris at pH 7.4 and 0.05% Nonidet P40, secondly with C219 at 25 ng ml$^{-1}$ or with 265/F4 at 25 ng ml$^{-1}$ diluted in NGA buffer and finally with $^{125}$I-labelled rabbit antimouse Ig diluted 1:1000 in NGA buffer before autoradiography.

### *RNA preparation, electrophoresis and Northern blotting*

Total cellular RNA was prepared from cells lysed in guanidine chloride by ethanol precipitation and phenol/chloroform extraction of proteins as described previously (Reeve *et al.*, 1989*a*). Fifteen to thirty $\mu$g of total cellular RNA, denatured by glyoxalation, were separated by electrophoresis through 1.4% agarose in 10 mM sodium phosphate buffer at pH 7.0 and transferred to nitrocellulose filters by Northern blotting.

### *First strand cDNA synthesis and amplification by PCR*

cDNA sequences were synthesised from 1–5 $\mu$g of total cellular RNA in the presence of 10 mM dithiothreitol, 6 mM $MgCl_2$, 40 mM KCl, 200 $\mu$M of each deoxynucleoside triphosphate, 2.5 $\mu$g of DNAase/RNAase free bovine serum albumin and 50 mM Tris buffer at pH 8.3 with 0.5 $\mu$g of random hexamer, pd(N)6 as primer in a total volume of 25 $\mu$l. After an initial exposure of this mixture to 65°C for 10 min, 20

units of super reverse transcriptase were added to initiate DNA synthesis and the reaction mixture incubated at 42°C for 1–2 h. Five to ten μl of this mixture was then used for amplification of specific DNA sequences in the presence of 2 mM $MgCl_2$, 50 mM KCl, 200 μM of each deoxynucleoside triphosphate, 5 μg of DNAase/RNAase free bovine serum albumin and 20 mM Tris buffer at pH 8.3 together with 50 pmoles of each primer in a total volume of 50 μl. The reaction was initiated by addition of 2 Units of Taq polymerase (Cetus) and amplification proceeded through 30 cycles of 95°C for 1 min, 55°C for 2 min and 72°C for 2 min. The sequence of the reverse primer (CGAGCCTGGTGGTC-AGT) was common to all mouse *mdr* genes, representing bases 1930–1913 in *mdr*1a otherwise termed *mdr*3 (Hsu *et al.*, 1989, 1990) and 2449–2432 in *mdr*1b otherwise termed *mdr*1 (Gros *et al.*, 1986*b*). The sequences of the forward primers were specific either to the *mdr*1a gene (AGCATC-TGTGGACCACATG) or to the *mdr*1b gene (TGCATACA-ACCAGTGTTTG) representing bases 1485–1503 in *mdr*1a and 2161–2179 in *mdr*1b. The amplification products were separated by electrophoresis in 2% agarose in a buffer containing 20 mM sodium acetate, 40 mM Tris and 0.2 mM EDTA at pH 8.3 and visualised by u.v. in the presence of ethidium bromide. For further identification of the sequences, the amplified DNA products were blotted onto nylon filters.

### *Preparation of radiolabelled probes and hybridisation*

The pcDR1.3 probe for the mouse λDR11 gene coding for P-glycoprotein (Gros *et al.*, 1986*a*) was generously provided by Dr James M. Croop (Centre for Cancer Research, Massachusetts Institute of Technology, USA). This probe was oligolabelled with $^{32}$P-CTP using Klenow fragment DNA polymerase and separated from unbound label by centrifugation through Sephadex G-50. Following prehybridisation for 1 h at 65°C with the hybridisation mixture containing 1% SDS, 6 × SSC, 0.5% salmon sperm DNA, filters were exposed overnight at 65°C to the labelled probe diluted in the hybridisation mixture and subsequently washed several times at 65°C in a mixture containing 0.1 × SSC and 0.1% SDS before being subjected to autoradiography.

### Results

The monoclonal antibodies MRK16, JSB-1 and C219 produced cell edge staining in 60–70% of cells of the human MDR cell lines, H69/LX4, LX20 and VR2 but not in the sensitive parent cell line, H69/P (Table II). The antibody, 265/F4 did not stain any of the parent or resistant human cell lines. By contrast, MRK16 and JSB-1 did not detect P-glycoprotein in any of the resistant mouse cell lines. C219 however still produced strong cytoplasmic and edge staining in the resistant variants, but not the sensitive parent cells of both the EMT6 (Figures 1a and 1b) and the RIF lines (Figure 1c). With 265/F4, no staining was visible in the resistant (Figure 1d) or parent EMT6 cells, but strong cell edge and cytoplasmic staining was evident in the resistant cells of the RIF line (Figure 1e). In each resistant cell line, staining with both C219 and 265/F4 was heterogeneous within the cell population, 60–70% of cells being positive. Staining in the less resistant lines, RIF/0.1 and RIF/0.2 (Figure 1f) appeared not to be at the cell edge, but predominantly in patches in the cytoplasm. Subcellular fractionation studies are now being undertaken to determine whether this apparent intracellular location of P-glycoprotein is genuine. If so it raises interesting possibilities with regard to the function of the protein in normal tissues and to the effect of the protein on cytotoxic drug distribution in resistant cells.

To obtain more quantitative analysis of the above immunohistochemical data, we performed whole cell ELISA assays on the mouse cell lines. These showed staining intensity with both C219 and with 265/F4 to increase in parallel with increasing resistance in the RIF cell lines (Table III). C219 also produced strong staining with the resistant EMT6 cells, but there was not such a close correlation between resistance and absorbance in these EMT6 lines. Differences in cell size and therefore in membrane surface areas in EMT6 sublines derived in different drugs (the VCR-selected cells appear larger than the ADM-selected cells) will also influence the amount of P-glycoprotein measurable per cell.

Western blot analysis was conducted on membrane proteins prepared from both human and mouse resistant cell lines. When probed with JSB-1, a single major band at a position corresponding to a Mr of around 160–180 kDa was seen in membranes prepared only from human resistant cells, but not from sensitive parent human cells or resistant mouse cells. By contrast, when probed with 265/F4, a single 170 kDa band was seen in membranes prepared only from the resistant mouse fibrosarcoma RIF cell lines and no such band could be seen in membranes prepared from the resistant EMT6 cells (Figure 2a) or from resistant human cells. With C219, a 170 kDa band and two additional protein bands of about 85–95 kDa were evident in membranes prepared from the resistant cells of both the EMT6 and the RIF cell lines (Figure 2b) and a single 170 kDa band in membranes from human resistant cells. However, when mouse resistant cell membranes were prepared in the presence of the protease inhibitors, aprotinin, leupeptin and pepstatin, rather than PMSF alone, only a single 170 kDa protein band was detected by C219 (Figure 2c). It would appear therefore that the smaller bands represent degraded fragments of the larger 170 kDa protein.

In order to identify which genes encoding P-glycoprotein were being expressed in the mouse tumour cell lines, total RNA was prepared from both resistant and parent cells of EMT6 and RIF cell lines and following reverse transcription, cDNA sequences were amplified by the polymerase chain reaction using primers specific for either *mdr*1a or *mdr*1b genes. Resolution of these amplified sequences on 2% agarose is shown in Figure 3. With the *mdr*1b-specific forward primer, it was possible to distinguish an amplified DNA transcript corresponding in size to the expected 289 bp in material obtained from all the RIF cell lines, but not in that from the EMT6 cell lines. The band stained only weakly with ethidium bromide in the parent RIF line and most strongly in the most resistant RIF 1.0 line. With the *mdr*1a-specific forward primer, a slightly larger amplified DNA sequence corresponding to the expected 446 bp was seen in material taken from all the resistant EMT6 cell lines. The band could also be distinguished, albeit weakly, in the parent EMT6. There was progressively increased expression of *mdr*1b in the resistant fibrosarcoma lines showing progressively increased levels of resistance, i.e. RIF/0.1, 0.2, 0.4 and 1.0. However

**Table II** Immunoreactivity of different monoclonal antibodies with cytospins prepared from sensitive and resistant human and mouse tumour cell lines

| *Cell line* | *C219* | *Monoclonal antibody* *MRK16* | *JSB-1* | *265/F4* | *Blank (mouse serum)* |
|---|---|---|---|---|---|
| H69/P | – | – | – | – | – |
| H69/LX4 | ++ | ++ | ++ | – | – |
| H69/LX20 | ++ | ++ | ++ | – | – |
| H69/VR2 | ++ | ++ | ++ | – | – |
| EMT6/P | – | – | – | – | – |
| EMT6/CR0.2 | + | – | – | – | – |
| EMT6/VR1.0 | + | – | – | – | – |
| EMT6/AR1.0 | ++ | – | – | – | – |
| EMT6/CR2.0 | ++ | – | – | – | – |
| RIF-1 | – | – | – | – | – |
| RIF/0.1 | +/– | – | – | +/– | – |
| RIF/0.2 | + | – | – | + | – |
| RIF/0.4 | ++ | – | – | ++ | – |
| RIF/1.0 | ++ | – | – | ++ | – |

Intensity of staining: –, negative; +/–, weak; +, strong; ++, very strong.

a
b
c
d

**Figure 1** Immunocytochemical staining with C219 (**a**, **b** and **c**) and with 265/F4 (**d**, **e** and **f**) of cytospins prepared from **a** EMT6/CR0.2; **b**, EMT6/VR1.0; **c**, RIF/0.4; **d** EMT6/VR1.0; **e** RIF/0.4 and **f**, RIF/0.2 (Original magnification × 622).

**Table III** Absorbance values obtained from whole cell attached ELISA assays showing the relative amounts of P-glycoproteins detected by C219 and by 265/F4 in resistant variants derived from the mouse mammary tumour cell line, EMT6 and from the mouse fibrosarcoma cell line, RIF-1

| Cell lines | *Absorbance at 405 nm per $10^5$ cells* with C219 | with 265/F4 |
|---|---|---|
| EMT6/P | 0.04 | nd |
| EMT6/CR0.2 | 0.32 | nd |
| EMT6/AR1.0 | 0.73 | nd |
| EMT6/VR1.0 | 1.00 | nd |
| EMT6/CR2.0 | 1.38 | nd |
| RIF-1 | 0.02 | 0.10 |
| RIF/0.1 | 0.07 | 0.28 |
| RIF/0.2 | 0.24 | 0.40 |
| RIF/0.4 | 0.42 | 0.83 |
| RIF/1.0 | 0.74 | 1.18 |

Values shown are the mean of data from 2–3 separate experiments. nd = not determined.

**Figure 2** Immunodetection of P-glycoprotein on Western blots of membranes prepared from sensitive EMT6/P and RIF-1 and drug resistant EMT6/AR1.0 and RIF/1.0 cell lines. Filters were probed with **a**, 265/F4 or with **b** and **c**, C219. Fifty μg of membrane protein were loaded per track. Membranes were prepared in the presence of **a** and **b** PMSF alone or **c** a mixture of aprotinin, leupeptin and pepstatin.

expression of *mdr*1a was evident only in the more resistant RIF cell lines (Figure 4).

Southern blots were made from some of the gels containing the PCR products and were hybridised with the mouse-specific pcDR1.3 probe. Following washing to high stringency, autoradiographs revealed a major radioactive band corresponding in position to that of the 289 bp sequence amplified with the *mdr*1b-specific primer. No radioactive band was evident at the position corresponding to that of the 446 bp sequence amplified with the *mdr*1a-specific primer. This was not unexpected since the *mdr*1a sequence amplified was from a region which shares little homology with the transcript of the *mdr*1b gene and the probe, pcDR1.3 was derived from a mouse cell line expressing only the *mdr*1b gene (Gros *et al.*, 1986*a*). The amplified sequences were also oligolabelled and themselves used to probe Northern blots of RNA prepared from resistant cells both of the EMT6 and of the RIF cell lines. The *mdr*1a probe recognised a band of RNA of size similar to that detected by the mouse-specific pDR1.3 probe, i.e. around 5 kb. This was present in all the resistant EMT6 cell lines and also in the more resistant RIF cell lines. The *mdr*1b probe recognised a

**Figure 3** Separation of PCR amplified cDNA sequences transcribed from total RNA prepared from EMT6 and RIF-1 cell lines. cDNA in each sample was amplified in the presence of primers specific either for the mouse *mdr*1a gene **a** or the mouse *mdr*1b gene **b**. Amplified sequences were resolved on a 2% agarose gel and visualised by ethidium bromide fluorescence. The DNA markers are from HaeIII-digested ΦX174-RF DNA.

**Figure 4** Separation of PCR amplified cDNA sequences transcribed from total RNA prepared from RIF-1 cell lines with increasing levels of resistance left to right, sensitive parent RIF-1, low resistance RIF/0.1 and RIF/0.2, and higher resistance RIF/0.4 and RIF/1.0. cDNA in each sample was amplified in the presence of primers specific either for *mdr*1a or *mdr*1b.

similar sized band of RNA, but this was present only in the RIF cell lines.

### Discussion

MRK16 and JSB-1 stained resistant variants of the human tumour cell line only and failed to recognise P-glycoprotein in the mouse MDR cells. Selectivity of MRK16 for the human *mdr*1 protein has been indicated by previous work (Thiebaut *et al.*, 1989). C219, which recognises a sequence present in all P-glycoproteins so far characterised, stained resistant cells of both human and mouse tumour cell lines.

265/F4 appeared the most selective of the four monoclonal antibodies, failing to recognise human *mdr*1 P-glycoprotein on cytospins or Western blots and producing differential staining between the resistant mouse tumour cell lines. It seems that this antibody can detect a protein the amount of which correlates with the level of multidrug resistance in RIF cell lines, but which is not present in the resistant EMT6 lines. The results of PCR amplification of specific *mdr* transcripts indicate that in the highly resistant RIF cell lines two separate genes, *mdr*1a and *mdr*1b are expressed whilst in the resistant EMT6 cell lines only one gene, *mdr*1a, is expressed. It seems highly likely therefore that the basis of the differential staining with 265/F4 resides in its selective recognition of the *mdr*1b gene product.

Whether it is the actual primary amino acid sequence of the 265/F4 epitope that is lacking in the *mdr*1a protein or whether the epitope is simply inaccessible to the antibody due to secondary modifications such as glycosylation or the way in which the protein is inserted in the membrane of the EMT6 cells is not entirely clear. The latter suggestion would however seem unlikely in view of the findings from the Western blot analysis that 265/F4 retains its ability to bind to the *mdr*1b protein that has been solubilised in SDS and presumably unravelled from its membrane environment.

It is difficult to understand what structural requirements may be necessary for 265/F4 recognition of different P-glycoproteins. It has been reported that 265/F4 can produce positive staining in human lung carcinoma xenografts (Volm *et al.*, 1988*a*) and in frozen sections of human renal cell carcinoma where the distribution of staining paralleled that produced by C219 (Bak *et al.*, 1990). Yet in our resistant human small cell lung carcinoma lines which stain strongly with other anti-P-glycoprotein antibodies, we were unable to detect any reactivity with 265/F4 either in cytospins or on Western blots. Similar negative findings have been reported with 265/F4 on resistant human breast carcinoma (Lathan *et al.*, 1985). There may be differences between P-glycoproteins expressed in different human tissues that will influence antibody recognition. For instance the disposition of the proteins within the membrane or post-translational modifications to the proteins may differ. Certainly the *mdr*1b P-glycoprotein expressed in mouse tissues appears to be glycosylated to different extents in uterine tissue and in MDR cell lines (Greenberger *et al.*, 1989). Surface modifications might well be significant for 265/F4 recognition since the antibody, raised originally against whole intact resistant cells, probably recognises an external epitope.

Despite the uncertainty surrounding the interpretation of the antibody recognition of the P-glycoprotein isoforms, it is clear from the data obtained by PCR that only *mdr*1a is expressed in the mouse resistant mammary tumour cell lines whilst both *mdr*1a and *mdr*1b are expressed in the resistant fibrosarcoma cell lines and that expression of *mdr*1b precedes that of *mdr*1a during the acquisition of resistance. Expression of both *mdr*1a and *mdr*1b within a single cell has been reported previously in other mouse drug resistant cell lines. e.g. of the macrophage-like type (Hsu *et al.*, 1989). Here also the *mdr*1b gene alone was expressed in the lower resistance lines and higher resistance was associated with a switch to *mdr*1a expression. The functional significance of these two different gene products is not yet clear but it has been suggested that the *mdr*1a gene product may act as a more efficient pump to expel drug from the cells. Although both can confer multidrug resistance in transfection experiments, it has been shown that there are striking qualitative and quantitative differences in the drug resistance phenotype conveyed by these two genes (Devault & Gros, 1990). In these particular experiments, cell clones expressing the *mdr*1b gene were more resistant to adriamycin and to colchicine than those showing an equivalent level of expression of the *mdr*1a gene. In our cell lines, the *mdr*1a protein appeared more susceptible than the *mdr*1b protein to degradation following disruption of cellular structure, smaller fragments being detectable on Western blots. However it has been reported that in intact cells, rates of degradation of these two gene products are similar (Cohen *et al.*, 1990). The different gene products have been detected in different amounts in different normal tissues in the mouse (Croop *et al*., 1989), very high levels of the *mdr*1b gene product being found in the uterus of the pregnant mouse where it may play an important role during gestation (Arceci *et al*., 1988; Greenberg *et al*., 1989). The possibility is that they may therefore be involved with the transport of quite different endogenous substances. It is thought that each of these *mdr* genes is regulated in a tissue-specific manner. An understanding of the factors involved in this differential regulation may well therefore be important in designing cancer chemotherapy strategies (Croop *et al*., 1989).

We are grateful to Professor W. McGuire for maing antibody 265/F4 available to us. Similarly antibody MRK16 was kindly supplied by Professor T. Tsuruo. We also acknowledge the help of Dr P. Rabbits and Ms Jenny Douglas with the molecular biology techniques and Mrs Karen Wright with the cell culture. This work was supported by a grant from the Cancer Research Campaign.

### References

ARCECI, R.J., CROOP, J.M., HORWITZ, S.B. & HOUSMAN, D. (1988). The gene encoding multidrug resistance is induced and expressed at high levels during pregnancy in the secretory epithelium of the uterus. *Proc. Natl Acad. Sci. USA*, **85**, 4350.

BAK, M., EFFERTH, T., MICKISCH, G., MATTERN, J. & VOLM, M. (1990). Detection of drug resistance and P-glycoprotein in human renal cell carcinomas. *Eur. Urol.*, **17**, 72.

BARRAND, M.A., TSURUO, T. & TWENTYMAN, P.R. (1990). Differences between monoclonal antibodies in immunohistochemical detection of P-glycoprotein in human and mouse multidrug resistant cell lines. *Br. J. Cancer*, **62**, 510.

BARRAND, M.A. & TWENTYMAN, P.R. (1991). Variations in P-glycoprotein expression in response to drug treatment in mouse tumour cell lines. *Br. J. Cancer*, **63** Suppl.XIII, 12.

BARRAND, M.A., WRIGHT, K.A. & TWENTYMAN, P.R. (1991). Differences in the early appearance and localisation of two P-glycoproteins encoded by the genes *mdr*1a and *mdr*1b following drug exposure in mouse tumour cell lines of different tissue origin. *American Association for Cancer Research Special Conference on Membrane Transport in Multidrug Resistance, Development and Disease*. Banff, Canada.

COHEN, D., YANG, C.-P.H. & HORWITZ, S.B. (1990). The products of the *mdr*1a and *mdr*1b genes from multidrug resistant murine cells have similar degradation rates. *Life Sci.*, **46**, 489.

CROOP, J.M., RAYMOND, M., HABER, D. & 4 others (1989). The three mouse multidrug resistance (*mdr*) genes are expressed in a tissue-specific manner in normal mouse tissues. *Mol. Cell.Biol.*, **9**, 1346.

CUMBER, P.M., JACOBS, A., HOY, T. & 4 others (1990). Expression of the multiple drug resistance gene (*mdr*-1) and epitope masking in chronic lymphatic leukaemia. *Br. J. Haematol.*, **76**, 226.

DEVAULT, A. & GROS, P. (1990). Two members of the mouse *mdr* gene family confer multidrug resistance with overlapping but distinct drug specificities. *Mol. Cell. Biol.*, **10**, 1652.

GEORGES, E., BRADLEY, G., GARIEPY, J. & LING, V. (1990). Detection of P-glycoprotein isoforms by gene-specific monoclonal antibodies. *Proc. Natl Acad. Sci. USA*, **87**, 152.

GEORGES, E., TSURUO, T. & LING, V. (1991). Topology of P-glycoprotein as determined by epitope mapping of MRK-16 monoclonal antibody. *Proceedings of the American Association for Cancer Research Special Conference on Membrane Transport in Multidrug Resistance, Development and Disease*. Banff, Canada.

GREENBERGER, L.M., CROOP, J.M., HORWITZ, S.B. & ARCECI, R.J. (1989). P-glycoproteins encoded by *mdr*1b in murine gravid uterus and multidrug resistant tumor cell lines are differentially glycosylated. *FEBS Lett.*, **257**, 419.

GROGAN, T., DALTON, W., RYBSKI, J. & 10 others (1990). Optimization of immunocytochemical P-glycoprotein assessment in multidrug-resistant plasma cell myeloma using three antibodies. *Lab. Invest.*, **63**, 815.

GROS, P., BEN NERIAH, Y., CROOP, J.M. & HOUSMAN, D.E. (1986*a*). Isolation and expression of a complementary DNA that confers multidrug resistance. *Nature*, **323**, 728.

GROS, P., CROOP, J. & HOUSMAN, D.E. (1986*b*). Mammalian multidrug resistance gene: complete cDNA sequence indicates strong homology to bacterial transport proteins. *Cell*, **47**, 371.

HAMADA, H. & TSURUO, T. (1986). Functional role for the 170- to 180-kDa glycoprotein specific to drug resistant tumor cells as revealed by monoclonal antibodies. *Proc. Natl Acad. Sci. USA*, **83**, 7785.

HSU, S.I.-H., COHEN, D., KIRSCHNER, L.S., LOTHSTEIN, L., HARTSTEIN, M. & HORWITZ, S.B. (1990). Structural analysis of the mouse *mdr*1a (P-glycoprotein) promoter reveals the basis for differential transcript heterogeneity in multidrug-resistant J774.2 cells. *Mol. Cell. Biol.*, **10**, 3596.

HSU, S.I.-H., LOTHSTEIN, L. & HORWITZ, S.B. (1989). Differential overexpression of three *mdr* gene family members in multidrug-resistant J774.2 mouse cells. Evidence that distinct P-glycoprotein precursors are encoded by unique *mdr* genes. *J. Biol. Chem.*, **264**, 12053.

JURANKA, P.F., ZASTAWNY, R.L. & LING, V. (1989). P-glycoprotein: multidrug-resistance and a superfamily of membrane-associated transport proteins. *FASEB J.*, **3**, 2583.

KARTNER, N., EVERNDEN-PORELLE, D., BRADLEY, G. & LING, V. (1985). Detection of P-glycoprotein in multidrug resistant cell lines by monoclonal antibodies. *Nature*, **316**, 820.

LATHAN, B., EDWARDS, D.P., DRESSLER, L.G., VON HOFF, D.D. & MCGUIRE, W.L. (1985). Immunological detection of Chinese Hamster ovary cells expressing a multidrug resistance phenotype. *Cancer Res.*, **45**, 5064.

REEVE, J.G., RABBITTS, P.H. & TWENTYMAN, P.R. (1989*a*). Amplification and expression of *mdr*1 gene in a multidrug resistant variant of small cell lung cancer cell line NCI-H69. *Br. J. Cancer*, **60**, 339.

REEVE, J.G., WRIGHT, K.A., RABBITTS, P.H., TWENTYMAN, P.R. & KOCH, G. (1989*b*). Collateral resistance to verapamil in multidrug-resistant mouse tumour cells. *J. Natl Cancer Inst.*, **81**, 1588.

ROCKWELL, S.C., KALLMAN, R.F. & FAJARDO, L.F. (1972). Characteristics of a serially-transplanted mouse mammary tumor and its tissue-culture-adapted derivative. *J. Natl Cancer Inst.*, **49**, 735.

SCHEPER, R.J., BULTE, J.W.M., BRAKKEE, J.G.P. & 8 others (1988). Monoclonal antibody JSB-1 detects a highly conserved epitope on the P-glycoprotein associated with multi-drug-resistance. *Int. J. Cancer*, **42**, 389.

SCHINKEL, A.H., ROELOFS, M.E.M. & BORST, P. (1991). Characterization of the human MDR3 P-glycoprotein and its recognition by P-glycoprotein-specific monoclonal antibodies. *Cancer Res.*, **51**, 2628.

THIEBAUT, F., TSURUO, T., HAMADA, H., GOTTESMAN, M.M., PASTAN, I. & WILLINGHAM, M.C. (1989). Immunohistochemical localization in normal tissues of different epitopes in the multidrug transport protein P170: evidence for localization in brain capillaries and crossreactivity of one antibody with a muscle protein. *J. Histochem. Cytochem.*, **37**, 159.

TWENTYMAN, P.R., BROWN, J.M., GRAY, J.W., FRANKO, A.J., SCOLES, M.A. & KALLMAN, R.F. (1980). A new mouse tumor model system (RIF-1) for comparison of end-point studies. *J. Natl Cancer Inst.*, **64** 595.

TWENTYMAN, P.R., FOX, N.E., WRIGHT, K.A. & BLEEHEN, N.M. (1986). Derivation and preliminary characterisation of adriamycin resistant lines of human lung cancer cells. *Br. J. Cancer*, **53**, 529.

TWENTYMAN, P.R., REEVE, J.G., KOCH, G. & WRIGHT, K.A. (1990*a*). Chemosensitisation by verapamil and cyclosporin A in mouse tumour cells expressing different levels of P-glycoprotein and CP22 (sorcin). *Br. J. Cancer*, **62**, 89.

TWENTYMAN, P.R., WRIGHT, K.A. & FOX, N.E. (1990*b*). Characterisation of a mouse tumour cell line with *in vitro* derived resistance to verapamil. *Br. J. Cancer*, **61**, 279.

VAN DER VALK, P., VAN KALKEN, C.K., KETELAARS, H. & 8 others (1990). Distribution of multi-drug resistance-associated P-glycoprotein in normal and neoplastic human tissues. *Ann. Oncol.*, **1**, 56.

VOLM, M., BAK, M., EFFERTH, T., LATHAN, B. & MATTERN, J. (1988*a*). Immunocytochemical detection of a resistance-associated glycoprotein in tissue culture cells, ascites tumors and human tumor xenografts by Mab 265/F4. *Anticancer Res.*, **8**, 531.

VOLM, M., BAK, M., EFFERTH, T. & MATTERN, J. (1988*b*). Induced multidrug-resistance in murine sarcoma 180 cells grown *in vitro* and *in vivo* and associated changes in expression of multidrug-resistance DNA-sequences and membrane glycoproteins. *Anticancer Res.*, **8**, 1169.

VOLM, M., BAK, M., EFFERTH, T. & MATTERN, J. (1989). Induced multidrug resistance in murine leukemia L1210 and associated changes in surface-membrane glycoprotein. *J. Cancer Res. Clin. Oncol.*, **115**, 17.